# Supplementary material for: Ionotropic receptors in the turnip moth Agrotis segetum respond to repellent medium-chain fatty acids
Source: BMC Biol. 2022 Feb 7;20:34. doi: 10.1186/s12915-022-01235-0 (PMC8822749; doi:10.1186/s12915-022-01235-0)
Supplement: Supplementary file 2 — Additional file 2. Amino acid sequences of AsegIRs. [file 12915_2022_1235_MOESM2_ESM.docx]

**Amino acid sequences of AsegIRs**

>AsegIR1.1

MILPTILFSLFLNVLGFDLDVANFITDYVLFKDLRYLCYFTCESPYYNTIVVHKLTKENIRVAVRRIDTSDRVDMVKVAYQHTAAVGMIVDGHCDNTPALLLQASSSKLFDAMHPWLVLSDTDDPDNCTQYIDDIFKPLNLSVDADITVVTRDGYNYTMIDVFNFGKIQGNQLEIALLGSWRPDEGLDILLKGYKYYNRWNFHNLTLRAITVIVEQPEVFYPEMLSEMGYTAGVAAMTKITSQMLNTIKEQHNFRFNYSIAGRWIGAPERNTTLAVTNSLLWEETDLSSTCARIFPNWLDWVDIYHPPTTNLQTKFYYLIPQKGVGQYENQFLTPMSDGVWGCAFLAGIACTLVLTVAARLENRPKSGWYAFFSTFAAICQQDFEDGVQLLEETFSSQGRKATLLVIGLTSMLLYNYYTSSVVSWLLNAAAPSIATLDGLISSDFELIFEDIGYTRGWLDNPGFFYYSGFTNLKEDELREKKVTKAKRSAAVLQTVKTGVELMRTGKYAFHTEPYTASQVISKTFEDAELCNLGALQMMLPAHVYIMAQKRSPYKEFFDWSLLRLTERGHVKAIRARFAGDMPACSGAQPRALALGQAAPAFLTLFLFAVLAYIILAFEILWQRVQLKKRAVSEAQKLPSPVIGL

>AsegIR1.2

MWFQAILTSMLACLVFGLDQNALKFSIEYFKDRDVKFICLLTCGKESSWTLQFSKLSTMAISGISIDDSLTNYKRVENCLQRKYYSVGVIIDATCNKCEDVLYFASQNMWLDSNHKWLILDDDEAEIESYNDTVINTILFDKDRNTTLIEVLDKLNISVDADIMMAKRDNSKYTLYDVYNYGKIQGGNMILHKVGTWDLYNGLRLDINLNGYKYYRRWDFQNITMKMVLVVQPVPKHFDPELLTGLETVPGVSMITQTSAAVLYVVAKMHNIRYTPTITDRWIGTYERNSSRVVSNSLYFREQDLSPVIRLLKSVQENNDVLNPPLTAIETRYYYRIPTVGPGKFENQFLRPLSPTAWWSVIAVSGLCAALLLLSALLEKRPSSVQYAVFSVVASICQQFFQDIDDGATKRISTARKVTILVTGLSCVLLYNYYTSSVVSWLLNGPPPSINSLKELLESPLELIYEDIGYTRSWLQTPSYYFNKRNAKIEDELRKKKVFNKKKDAPLLVDLVEGIKMVQRGGYAYHTEVNSANALISKTFSQDELCELGSLKSMEKTVLYAVLQKNSPYKEFMTWSLMRLAEQGIVSCIQIRRSSFEVKCEGSSPRALALGGAAPAFILLAGGYMLATVIMMVERMVYKMKLEHNIPQ

>AsegIR7d.1

MFVSLVRQNLYPSPPKMDFREKGARNHTINDLITEEPVSELGLLAVKIAFYNFEWRFLTMVMFNTLQAIGLKTFLMQYQQSVVVKLGRFLPARRSAVPQMVIFGEDSSEISSTLRWTVRSKYDSNGKYIIVCALLDQECDEQKIFQTLESLFILNVVLLKTSHKTNKSLAYSYDFVSHGTCKNNVPYQLNLSTTCENDKCFKELYAEKLMNFHKCRLVMSTIEQPPFMYLHNYTTSPSGIDGDIMKLAADMLNATLVLIKPEDGGDAGSFINNNWTGSLGDIYNGRTHASVCSTPITVNKYGNFQISFTYYSMDIVWSARLPTQKSSWEKLLSPLNIYMRIILLLFFIAIVFMNTICTSTIWRQMRRTFKIEPPKYSFLFFSWVLFLGLPILREPERKSFLVIIYTWIWFCFVIRSAYQAALINSLKQPAYLDNFETFQDVLNNGYPYGGSASLKEYYTDDRFIYNHWKVVDQARYYEILDDIMDGTTDFVLASNKEAVKHHVMRFNGTKQLQIIPEKIVNSPTVVYYKKFSPLTGPLNTVLRTALEGGFINRIHYKYLDHDKKLFQRVQSHRPEPLRMNHFTGSFVLLIIGWFVSTIYFIVEYVCGNLKD

>AsegIR7d.2

MAAKFAMQNFDLRYTTLLLFNSTFCYGVEVFLRLYHYNIVVNKSPFKPNSQTQRTRQFVLFSSDAADVELILDAIVEFEMDNTGKFIIVCESPVPNECDEQDIVDLCWNYRIVNIVFIRLEATAAISFTYYPVADGICHNLKPIQLDRNNQYTHTTYGEVFRNKLRNFNWCPITVSTFIQSPYMLNITNGIPIGADGDLLRILMYGMNASLKMMTPNRGFGWGWRHENGTWMGSLADVDEDLANFSMTSAAITLTRFSDFQISSSYYSSKVVWVTQPAQFQNVALKLLHPFETDMRIALVVSFFLVIICAFLVKTDTWTSMCSRLVDDQANKSIVFYAWMICMGQAVIKLPTRSSFLQMALIWVWYCFLIRTAYQVYLISSLKGKFYETEFQTIEDVIDARFPFGGGVALKDYYIDYPIVYNNWKNIDSPQILPTALNITKGMDFVLAMNMDSAQMVIKSKRISLHILPQPVITSPTVIFFKKYSPLSE

>AsegIR7d.3

MVRLITVLSLLHLQFFSRSATSELVKTAVNITNTYYNLHFTTTVIWKLNDSKYVNDFLLSYPGSVVLSPWSIFNDSKIRDINETVGFKQTIYFANNLYDYEIIMQLINEVIRFPIRFILVLENAVKTNDEISDFIEVTSRNDQADLILISAYETGEVTLSTFFPYSDGCCSNYTPIFLKYGDNMFPKKFSNFYQCPIRTALLEYYPYVTVDVENGTITSVGGYDGKVLMIILNKLNASLEVTSAYNNSVFGSYVNGTATGSIGDLATEKADILIPADILTEKRYTVTLPSHTYHTVDIRWVGRRQREVSDWLKFIIPDTTNFTYLHGLVYISFLITAMLVRKCKPDMTSATNRILYQSFIILLGQSAKFVTKSWLLNFLFVLWIWFCFFFRIDYQADLVDALQTLDLEPPFASIEDAVTKVDGYGGVEVVVEYYKDTPLEHNYKVIPMNELRTYIKRIFEGENFLLATDVALVRSVEPYVQILEKRISATGASFYMRPGWPAAKDVDDVIFSLVEAGFIENLLSDDNNQRWLLNRKDDNDAYTPKGISLSKISTLFYALTVMWGICFIVLLAEIKHHKNKFKKTGW

>AsegIR8a

MSFYYLFLLIFLINLGCVISELSLRFVFIVETQEQDLTHNIGKALKLSETVRPDVKVDDAIVPLDREREEESYRILCSAVSKGASMIIDLSWSPWTMAEDLASETGVPLVRTLLGSQQLVKALDEHLESRNATDAAIILESESDVDQTLYELLGVSNIRVWVHAGLTRDSAKALKTMRPEPSFYIIVGVNGFVMDTYRRAVKEKLVRRNYRWNLVLTDYSTPDVSQLVLPTVMLQADQVECCKLMKREDCTCPSDFQRKQYILNALIQYIAETYSKLERDLSLTTSTIDCEDPQPLMNSTRERLFRQFGEDADMSNETIFFWDVERSGLFLRSRFILSMYKPDEGQQIIANWSADDEYKLLPGVELEPLKMFFRIGTAPAVPWTLVKIDPNTGEQMFDEDGQPLYEGYCVDLIARLSETMSFDYEIVSPKSGDFGKKLPNGTWDGVVGDLMRGETDIAISALTMTAEREEVIDFVAPYFEQTGILIVIRKPIRKTSLFKFMTVLRTEVWLSIVAALVLTGFMIWLLDKYSPYSARNNPDAYPYPCREFTLKESFWFALTSFTPQGGGEAPKALSGRTLVAAYWLFVVLMLATFTANLAAFLTVERMQTPVSSLEQLARQSRINYTVVEGSSVHQYFINMKFAEDTLYRVWKEITLNATSDQAQYRVWDYPIREQYGHILLAINASEPVPDAKTGFQQVNEHTDADFAFIHDSAEIKYEVTRNCNLTEVGEVFAEQPYAIAVQQGSRLQEDLSRALLELQKERFLEQLASKYWNESARQACPDADESEGITLESLGGVFIATLFGLGLAMITLAWEVFYYKRKEKNKVQAFNAKPEKPAFETKTTLETKVAESMAKLRRRGKVGKKGNVAKTVTIGDSFKPVAEKGVSYISVFPKDYRP

>AsegIR21a

MVEYYPSQAIINAQYKVESKREIKNIEIFENENSNNTSEEVKWRQFNQEENDDEDGDIHKRALDPVFHGHPKTREELWNERFLNQTLTFDQTPSLVHLLHNISLTYLKDCTPVILYDSLVKSKESYLVENLLKGFPMSYVHGYITDDGELVQPQLLHANTDCQHFILFLTEIKLSAKILGKQPENKVIIIARSSQWAVQEFLASVNSRNFVNLLIIGQSFKEGEDATRESPYILYTHRLYTDGLGASQPVVLNSWTHGKFSRDVHLFPPKMTKGYAGHRFVVAAANQPPYVFRRIKSDLDGGNPRVVWDGVELRLVKLLAERNNFSIEIIEPHEPNLGPGDSVAKEVKMGRADIGIAGIYLTNDRLTEMDVSLPHSQDCAVFVTLMSTALPRYRAILGPFHWHVWVALTFTYLFGMFPLAFSDKHTLRHLLHNSGEIENMFWYVFGTFTNCFTFLGKNSWSKTNKITTRLLIGWYWIFTIIITSCYTGSIIAFVTLPVFPETVDTIKQLLAGFYRVGTLDRGGWEKWFLNSSDPDTAKLLKKLEFVPNVEAGIRNTTKAFFWPYAFLGSKAELEYIVQANFTATKSKRAVLHISNECFVPFGITLAFPNNSVYSAKLNFDISRMIQSGLIDKITDEVRFEMQRSLTGKLLAAGSGVIKIPSAEEKGLSLEDTQGMFLLLGAGFLIAGTALISEWMGGFTRKCRFTRRIDTPISINSREHLIPTPRTNIESEIKIIGDTDSTLNFDSRPSTARSTETLDGQVINVSEENIDVHNTFNVGRFDSRRSSSLDLDREVREIFEKDMKRRRFTTHDMESLDENGSTVSRAAFGDPVKHDKIP

>AsegIR25a

MGDSSYNERHKIVVVTKMSAFSIFFFLLYLFRVAFGQTTQNINVLLINEESNALAEKAFEVAKEYVRRNPSLGLAVDPVIVVGNRTDAKSFLENVCRKYNDMLLAKKTPHVVLDFTMTGVGSETIKSFTEALGLPTISGSFGQVGDLRQWRSLNANQTRFLLQVMPPADILPEAIRAVVTKQDITNAAIIFDEFFVMDHKYKSLLQNIPTRHVITPVKSFEANEIKTQLESLRNLDIVNFFIVGSLRTIKNVLDAADKNQYFGRKTAWFALSLEKGDISCGCKNATIVHIRPTPDANSRDRLGKIKTTYSMNGEPEITSAFYFDLSLRTFLAIKSLLDSGKWPNDMKYITCDDYDGKNTPNRTLDLKTAFQEIKETPTYAPFFIPQDDPMNGRSYMEFSTDLLAITVKDGASISSHSLGSWKAGLSSNLTLTDPNNMSNYSAQLVYRIVTVEQKPFIIRDDKAPKGFKGYCIDLIEEIRLIVKFDYEISLAPDGNFGIMDENGNWNGIIKELVDKKADIGLSSLSVMAERENVVDFTVPYYDLVGITIMMKLPRTPTSLFKFLTVLENDVWLSILAAYFFTSFLMWVFDKWSPYSYQNNREKYKEDEEKREFTLKECLWFCMTSLTPQGGGEAPKNLSGRLLAATWWLFGFIIIASYTANLAAFLTVSRLDTPIESLDDLSKQYKIQYAPLNGSAAMTYFQRMANIEEKFYEIWKEMSLNDSLKEVERAKLAVWDYPVSDKYSKMWQAMEEAVLPNTIEEAIQRVRDSKSSSEGFAWLGDATDVKYHVMTSCDLQSVGDEFSRKPYAIAVQQGSPLKDQFNNAILQLLNKRKLEKLKEIWWNNNPEAMKCEKQDDQSDGISIQNIGGVFIVIFMGIGLACVTLGVEYWWYKWRKRPVVGDVVHVTQVEPAKSSRTNVDKQGDGFNFRGRNLGLNFSKPKF

>AsegIR31a

MLTQAVVDFFKHKVVSSIIVLACWSPIDQMKFTRQLSQNGMTATVSCDAAILDHIRHHYLQGVLYITHEHDEMPMFKKLKKTHFMMKYKWLILGDHVPDVLRKIRYDSDIAFLKWESGYFETKESTAISTESSTPIHIYDAYVHPRDGISINPWAHWTDSSGLVLTHERERILRRLDLKRYPLRIATPVGHYSSDKYDGTFVDFLEDESMSDQDPGIRSGYGTAMLLTEVVNAQDVLIENELWAAEINNNSMYVMVSTGEADISGAILRILFERTFTLDYVMPIWPFRVGFTYLAERESSSNMYLEPFSPAVWWSCLAMMVILAMVEWITAKSPKEKDGALYTVLTTWLQQDASAVPEGGSGRFAFTVLSVSAMLVHAYYTSAIVSALMSTGRGGPDSLKALGDSKYAIGSEDYDYMRYLFFDVKTTWDDLEYLKKKKMTSNFYQELERGVELIRQGSTAFHSEYNQIYPHFKTFSDDQICKLQHVDTIPESLTWIQSTKDGQWTEVLRTAGGWLLETGLGKRLVARLRVPQPPCRASLLAERVKLGDIAPLLALTVFGGFLSVVLLGVEILVAKTRSKRLREGDERGLEDVDDVSEVVEYDVNNLDP

>AsegIR40a

MNLIQKILLIFLSVNSAFGFFDIRDITSDVLTKLPKDFSIAVKDIAQALPSKSITIVRGNSTAIRSQDLLELLRMLSRHDVLTTNLNIATNENKQKYYKFLKKALDVSDQRTSLILCEPYECENILFELTENNLIHNMILYFFYWPYGPVSETFLMTIKEAMRVAVITNPRESVFRIYYNQGTPNRLNHLTLVNWWAGSLYKSPVLPPPDKVYDDFKGRVFEVPVLHAPPWHFVKYNNDNTITVTGGRDDKLLALLAKKLNFKYKYYDPPDRSQGSSISSNGTFKGTLGLIWKRQADFFLGDVTMTWERLQAVEFSFMTLADSGAFLTHAPAKLSETLAIIRPFQWEVWPLVFATLLVTGPALWIVIAAPSLWQKRKCDQLGLFSSCCWFTTTLFLRQSSSKEPSSTHKARLVSVLISLGATYVIGDMYSANLTSLIAKPSRERPIGTLPALEEAMRDYGYELVVESHSSSLAILENGTGVYGRLANLMRRQRIQRVRNVEMGVRLVLSRRRVAVLGGRETLYYDTERFGSHNFHLSEKLYTRYSAIALQIGCPYLETFNNVVMTLFEAGIIAKMTTDEYKNLPEQARRSDPVTESDKQGGEVMGDSAAASSQAPQGESTKGLQPVSLRMLRGAFCLLGIGYLLAAISFGVEIQLHRRSKRRREPPPNEHRKAQNLLVLGKSIMLFKRGCKKVCTTVYTGIDEALGPEVKD

>AsegIR41a

MLFPTSSSLLPLELLLNTIIRQYLESAYCITIISDVPLTFSWSKNFISLTPNKENLVEQIYNVSEMGCSDYIVRMQHPENFMTAFETVVHTAKVRRSDRKIIFLPYDEEYNEGYEIDLPSLVFSMKGSTYVANMLMIVNVASNTTDCKTFDLITHQFVGPDEQTHLPKYLDRWDSCSQEFENQANLFPHDMSNLYGKTLKVACFTYKPYALLDIDPAIEPSGRDGVEVRIVDEFCRWINCTVEVVKEDVDQWGEIYPNETGGIGVLGSVVEDRADVGISALYSWYEEWRVMDFSVAGVRTAITCIAPAPRLLSSWEMPLMPFTWYMWIAVAFTYIYASTGILTAQGCSTTSYPFLKTFGMMIGQSQYQNTESHSWKMRSVTGWLLIAGLILSSAYGAGLASTFTVPRYEPSIDTAQDIVDREMEWGATHDAWIFSLTLSTEPLVKQLVGQFRIYSFDVLKEKSFTRSMAFSIEKLPAGNFAIGEYVTQEAVLDMMIMLEDFYYEQCVLMMRKSSPYTEKVSQLVGRLHQSGLLLAWETQVALKHLNYKVQVEVRLSRNKNDVGSTEPLNLNNIVGVFIVYAIGLTISTAFFVGELYVYHRRTKNELVHE

>AsegIR60a

MSVKQIILVILLLVHNSNEVVNPHGPTVINDYTKCIIKVMENEFEEPGILIFANTNNNVSTSVSRIRTELLKKINENIKYSVEIMSPEKEVEICDNDNHNLGVLHVDNYVAIPTASYFVIIIDSYKDFSFLASKLIRSRSWNPFAKFIILLFNFFENDNNNIEYVEKVLSCLFKYNAINIIIAVPQANNFRNAIIYSWKPYDPPKYCGYFNETAKDRLVVQNMCERGVLKYAKDVFDNKIPRDMKGCVIEIIALQRHPFISDDPFEANIEKRMINEMLQRYNMKARYHFVEGFRGERERVGEWNGALKKLASKRGHLLLGGIFPDFDVHEDFETTITYLADAYTWVVPRAHRSAAWVALVIIFKKFVWLTVICGFFICGITWKIIGKLSGDTEYNSSLGHCLLNTWITILGFVAYLHPAKESLRVFFVFLNIYCILFLTAYQTKLFQVLTDPSYEYQIQTVEELIDSGLKFGGFEELHDLFYNSSDPFDYRIGEQWTDIENITEAMIDVAVHRNFSLLCSRLELAHISSITPELSDTVGNYKYYTFTENVFSVPIETIALRGFPFILEFSTTITLFKQSGLNEALRDHFATFNERRRARQLRALLKEKSDVNPLSGQHLQGGFLALAFGYVSGTLTFIVEIVVKTGYVQKKLSQCKRKIKLLS

>AsegIR64a

HKMFNDHDILIASAKIDYNATYDITKSYPRIGMVIDTACGGWTSVLSISTSPFQGYSYIIVTENLSLTTETLSRYPVEVDSDVTVAHRMNGTFILYEVYNTGFNFKGKYNVRIVGSWDTSLCIKDSKRWDLQGAFVKTAVVILSSPWLANQTVEQYMEKPIKSQIEVDTVHRMKFFTILKFMRDMYNISYDLHRVSTWGYKRNGSFDGMVNALYSGMAEIGGAPIFYRIDRGQEVQYISEVWMSRHSFVFRHPKYPGGFYTIYTRPLNVVVWYCVVTMLVVTAITLWAMLMVQAKRGDNEDSSFSLAGLVIWGAICQQGISINREATSTKLVIFTTFVYAVTLYQYYNATIVSSLLLEPPRNIRTLKDLLDSDLKAGSHDIVYDKDYFKRTTDPVAIELYHKKVATATHYNFFTPEEGIALVKKGGFAFHVETTYAFPLIKKTFTEREICETNLVQLYPLQRMGVVVRKNTPYKEHIAYAIRKMYEVGLPPRIQSEIDERMPECAHTPDSSVFCVRIREFSTPLLALMFGMISSVAIFMCEV

>AsegIR68a

MVVFKKMLKILIIGCLISGVKLQSFPILKDLHASKELDYLLIDLLNVLTREYEVTCVAIICDEVYLNVFAGPLFKRIVSVPYVMTVVEDYDDLLSPNFVTLESLRAARKEGCNVYVILLANGLQASRLLRFGDRHRILDTRAKYIMLHDFRLFRSELHYIWRRIVNIIFIKYHKKIIGVAKSKAWFELSTVPYPNPIKSIFVPRRVDIWKNENFHYKRPLFVDKTSNLNGEVLNVVYLGHVPSVVVTKNNGSNKIGGVEVEILHTLAEKMNFKPRPYQAPNADTHKWGQKQPNGSFSGLLGEMVNGGADIALGNLQYTPYHLELTDLSIPYTSQCWTFLTPEALTDNSWKTLILPFKLYMWIAVLLVLLITGTIFYGLAKNHMNLQEYKKNLRASNMKIDDQDQKPGLYLFGEIVNSILYTYGMLLVVSLPKLPVGWSIRLLTGWYWLYCILLVVSYRASMTAILANPAPRVTIDTLRELVESKVTCGGWGTQAKKFFEESLDEYSQRIGDKFETIDDPLLAADKVAQGVYAYYDNSDFLKYISVTRKNAFKDSKQNNATNTTEMTPRKDMQRNLHIMSDCVVNIPISIGFHKNSPLKTLADIYMRRVVEVGLVEKWLNDAMYSIRNLETNEEEVKALMNLKKLYGAFIALAIGYSLSAICLIGELIHWHFIVKRDPNFDKYALHMYYRQKNKKH

>AsegIR75d

MYSNTILWKKHQSLGLPLNPERGLAAEHYFLSDRRHVDMELINFILTYFLTKDLNMMTAFLCWTPAQVSELYRSSSRAGMRLALVSDFKHAPPMTPRGLFREAMLLDLACARSDLILEQASAARAFNYRYSWLLLHNSSSQRSTIEATLSASAILPDADVVWSSPETLVDVYSIKPGMPYLQTELGVARNSSRQELQILWGALPSVVSRRKDLRNVSMKGISVVTKPESFKSWWDLRDRQIDTFPKFTYPLMMLLTQDMNFRFDLRQVDVYGVVHNGTFDGLVGRLQRQEAELGIGSIFIRSDRMQAIDFISETCALTCAFIFRQPSRSAVSNVFLAPFSAGVWAAWALLLVALRKVRGSTRPETDLQQFTLPETFTFALGTLCQQGFYSTPAVTSVRLVMFSTLLAALFVFTAYSAKIVAILQTPSDALRTIDDLTRSPMLIGVQDTTYKKVYFLESPDESTQQLYRRKILPQGERAYLSEVEGIARVRTGFFAFQVERSSGYDIIKQTFTEREKCSLKEIEAFKLPMVAVPMRKHSGYRELFASRLRWQREVGLMDRERRMWLEPRPRCESSGGGFVSIGIIDVLPALQVLAMGALISVLLLATELGLHVMNKKTREYHHNHQLPRGKTMRAVMRRQN

>AsegIR75p

MLVFHSIKSMLVMNIISFTFFLFLQNFVQAKDETTINFIKLFIQNDQKPTHVIYGGLCWKKPVINKFVTEMSNIGVRTSASFKPKSKYQDHAIMYLTDLDCAQSRAVISYALSKELFQFTYRWLILTTTPELQQSTLALMENGPVLVDSDVVLAQRTADQFKMVEMHRPGLNGSMISTLRGYYNGSLVDVRPHRELYRRRKNMMGHPIIMSNVIQDSNTTRLHLPREDRLELQYDSITKACWSAAKIGFEMINATGRYIFSYRYGYKVNGQWSGMIADLYNNKADVGTNCVIFRDRFDVVTYTDLVAPMRMLFIFRQPPLAYVSNVFYLPFSTRVWVTIAVCTAIATVTIFLASKVEQVLTKASTQQQLDGGICDALLLTMSAVTQQGCYLEPRRAPGRMMVFVLFTALMALYAAYSANIVVLLQAPSDSIRSLPQLAGAKITLAANDVDYNHFVFNQSREPLYTSIRDRVFPENGKAKLYSLADGVERIRKGLFALHSVAEPVYRQIEATFLESEKCDIATVDYLVTFDSFTPVRKGSPYLELIRVVHKQIRESGIQSAIRKRYLVSKPHCITKISSFSSVGLMDMRPVLILKLYGVAVSVAIAIGEIAVHKLLNRYKKTSKVQMIKTVR

>AsegIR75p.1

MIVKTFLLISSALIVIVTCSESRIDAILSFVTLDNRPTSLLIPYLCWSQHALKSLAKSISSVGYGTATSLQYNRTEYDLQYILFLADLGCPGTDRFLVRASQEGFFKAPYRWLLLNHGDNSPALKDVDALVDSDLVVVKKISDEEYLFMEAYKVSEHSEVIYTNRATWKSGKTLNSDTKNNSLANKTIVYNTQNTSSINDQIFDTENTGIVFHEEQKVLPKITVTKYGWMEDYRSTNVLSSRRNDLRGHTLTMTNVITDSNETRQHMNDRLQLHQDSITKMSYAVVSICFQMLNATENLVFTHTWGYKDKHGNWQGIVDQLVKKEADLGTLTIFTQERMMVVDYIAMVGSTAVRFVFREPPLSYISNIFTLPFSGAVWLAIFICVLGCSIFLYIASKWEATMGMHPLQLDGSWADVLILIIGAVLQQGCTLEPRYGAGRCVTLILFVALTILYAAYSANIVVLLRAPSSSVRSLPDLLNSPLKLGASDFEYNRYFFKKLNDPIRKTIYEKKIAPKGKKPNYYSMKDGVERIRKGLFAFHMELNPGYRLIQETYQEDEKCDLVEIDYINEIDPWVPGQKRSPFKDLFKINFLRIRESGIQANVHQRLTVPRPRCSGHVSTFSSVGITDMYPAMLMTLYGMLLAPALLLVEILYKRLMTIRQQKRGLYAVDDHIPFRN

>AsegIR75p.2

MIFLNYYLKITEISYDLLFFCMLKISNMTPLFRALFFVFMLFEKEVYAKDPSWVHFIKSYVENEDKPTILILSDLCWDKNVIVSLANEISKIGSRSSTSLNIGSKYYYHDLLYLVDLDCPSAENVIALATKCNLFRSPYRWLVITAWSKHSNMAALWNSPILADSDVVLAAGTDSFLKLMELHKPSLNGSMISSVRGYYNGSLVDERPHRELFRRRRDVMGHTITMSTVIQDSNSTQYHLLMEDRLEPQYDSIAKICWMNVKLAFQMLNATPGHVFSYRWGYKVDGQWSGMIDDLYSGRADLGTNCVVSDLERLDVVSYTDNLAPFRVRFIFRQPPLPYVANIFSMPFSTNVWIAMSVCAVLSTATVFLAAKWEAKEGKGPTQLDSLGDAMLLTFSAIGQQGCVMEPRRLSGRMMVFVLFTALMALYAAYSANIVVLLQAPSDSIRSLPQLAGAKITLAANDVDYNHFVFKLFKDPVRELVYKRIDPEKGKKHFYDLNEGVERIRQGLFAFHSIVEPVYLRIEQTFLETEKCDLMEVDFLNSFDAFVPVRKDSPYLELLRVAFKQIRESGIQSAVSKRLQVPKPHCTSKMSSFSSVGLMDMKPVLIFMMYGVCLSVAIAVAEIVVFKLNPHRKASKVEITDSDVSNSA

>AsegIR75q.1

MLDTLFFNMKILTLITNIMCLKLCVSLSNPNSELQMIVDVIKAYDKPTAVTAKVCWDSATEIKLAKMLSNLDEPISILYLGDNKTIQQPYPNNHLLFIIDKHCKDTEFFLKQAGTNRMFSKSYRWLILGNTSQDEGIVASELNHVEISVDSEVITAQEIGNNDILLHTIYKLRADNEWNIEYYGTWSVDYGLNKSEDRMLSNMLRRKDFKGEPLTISVVIGDNRTKTDLLGLSNIFVDTIAKSSFRCIDPLYDFLNASRVIVFADTWGYLIDGTWNGMIGDIKNGRADLCGTVTYISIERMAILEYLTIPTPITAKFVFRQPPLSYQTNLFILPFSTGVWMSAGAFVLILGAILYINTKWDNKKYEKYNKQKMDQTCLPPTWGDITIFVLSAISQQGSSNELKGTLGRLVMFIVFLAFLFLYTSYSAYIVALLQSTSNRIRTLTDLLNSKLELGVEDVPYNRYYFSAAYSSKDPIKKAIFETKVAPRGKPNFMSIEEGVKAMQKKPFAFNMNIGTGYRIVSAFFQEHEKCGLQEIDFIQNNKPWLCSRKNSPFGEMFKIGYIRIQEHGLTDRENRLIYAKKPVCSVMGGSFDSVNMVDFYPVCLMLLYGMILAFILLGVEILVHRRQQKRESSRSNI

>AsegIR75q.2

MSNRYLKSYRKGIIQLTANADIILATMKELFFTVLLFSLSNGQDRQAAMIVDVIQSVGRPSSVIAKLCWTSTKIIQLHSLLTKEYIQFSAGDVINGDNAQFYDEEQHIVFLADLDCPDIDAYFQKNSFRNFFRAPFRWVFFRDPDKDDIVPEAVAKIDVLVDSEVLVLRSVDDTYEMHYIYKVGANTTWNTEFYGTWDTKKRFQKSPRFIEPTSLRRLDIDGYEISICYVLTNNKSVDHLTDGLDDHIDTITKVSFPTTNHLLDFLNAKRKYVFADTWGYRVNGTWNGMTGYLVRGEVEVGGSPMFFTFERVSIVDYISSPTPTRSKFVFQQPKLSYENNLFLLPFNSTVWYCTIALIFIIYLVLLMVTRWEWKKTSHMIESREKDSGVLRANVVDVIILIFGAACQQGSPSELKGSLGRVVMLVLFLALMFLYTSYSANIVALLQSSSSHIKTLEDLLHSRIKFGVHDTVFNRYYFSTATEPVRKAIYEKKVAPPGTTPRFMTMDEGVKQMRKGLFAFHMETGVGYKFVGKYFNEGEKCGLREIQYLQVIDPWLAVRKDTPFREMFKIGTKRIQEHGLQYRENRLMYEKRPKCSGGGSNFVSVSMVDCYPAVLILTYGAITALFLLALEILVFKREKLMHCMKHKSNH

>AsegIR76b

MSPFDHRPPNVSHGGDRRAHYAMAGIELIISSICNATFCEVPYNDTYKGPDALQAKEINFMNLAKEVNGKNLKVTTYNNTPLSWTEFHNGTVVGKGVAFIIMDILRKKFNFTYDVVEPRRNYEMGNKMNGDSIIGLVNTSKVDLAAAFLPTLIAYRERVSFSIDLDEGIWVMMLKRPKESAAGSGLLAPFNELVWYLVLAAVLTFGPCITFFTRVRSKLITDDEGVLPLKPSFWFVYSAFLKQGTNLAPEANTTRVLFVTWWLFMILLSAFYTANLTAFLTLSKFTLAIENPRDLYSKNYRWVASAGSSVEHVVKSDGEELYYLSAMISNGKAKFLSVTSDKDFLEAVKKGAVLVKEQTVVDHLMYNDYTSKKDVEESDKCTYVVAPNAFMKKQRAFAYPVGSKLKGLFDPVLTQIFQSGILDFLKRSDLPSTKICPLDLQSKDRKLRNSDLIMTYLVMVAGSGVAVAVFAAEIFIKRYISGKISTDKKGKRKKSKIGKKSTNYDDSRPPPYDSLFGKNPRFNVETTRTKIINGREYYVFETANGEKKLIPARAPSSFLYNRSDK

>AsegIR87a

MLAVLLFFIHFTSTISENPLLMTTGNSGQITKTAECVLKLSAKYFVEKKALSGSIVIININSYKSTTQGLLLQTIHSGIKYSIMVKDSFYPHANASHFPEKAKNYMLILEEKSELERNILQLNKLPTWNPLAKAIVFYQLKYNDTAEETATEFINELRDYKLFRTIVFIYDEYNDVVISYTWKPYSDTNCGGKCDSVYILDRCTNNTIYEFQKQHDLFPSNMKGCPLVAYAVIAEPYVMPPVSKLTNTSFEDAHVFAKGGEINLVKIISQFTNMSLITRTSEVQENWGEVFQNGTATGAFEVLRNESADLVIGNVEVTRILRKWFHPTVNYLQDEMTFCVPRAGQAPTWDNLVIIFQWTTWVATLFSFIIMGLMFHVFYYREHGNATKWPTNSLLMTFSMLLGWGATFEPKSPTFRILIFSWLCFSINMGISYESFLRSFLMHPRFEKQISTEADLILSGIPLGGREIYRSYFETNNASSFYLYRKYNSTTFSEGVRRAALQRNFAVVSSRRQAIYQDQKLGKGAPLIYCFPESNNMYKYGVAILTRRWFPMLDRFNNIIRSVSENGLIDKWMNELLIHSGNSEESSTIEPLSIQNLLGAFMFIGFMYAASIVIFLGELATGVIEKRRQSKKEVRGRLR

>AsegIR93a

MQLWIFSFVFLLAPVAAEDFPSLMTANASIAVVLDRQYLGEKYQTILDELKDYIKELARVELKHGGVIVYYYSWTAISLKKGFLAVFSVASCEDTWNLFSRTEEEELLLFALTEVDCPRLPSHSAITATFADPGEELPQLLLDLRTSNAFQWKSAIILHDDTLSRDMVSRVVQSLTSQIDDESASPVSVTVFKMKHEINEYLRRKEMHRVLSKLPVKYIGENFLAVVTSDGMTTMAETARDLVMSNTMAQWLYVISDTNAQNGNLSSLINDLYEGENVAYIFNMTENKPDCKNGIMCYCQELMDAFIAALDAAIQDEFDVAAQVSDEEWEAIRPNKLQRRDMLLKQMRQHIATKSRCGNCSTWRALAADTWGTTYRGLSDASDLASANLNGTTGVIDNINLLNVGFWRPIDAVKLEDVLFPHIHHGFRGKELPIITFHNPPWTILQRNESGAIVKYSGLIFDIVNQLAINKNFTIKVILASVLKKELANDTIADTMHGMDAKLTMIAISKGQGALGAAPFTVLSDPMPGINYTRPVSIQSYAFMIARPRELSRALLFLLPFTTDTWLCLGFAVILMGPTLYIIHRLSPYYEAKEITRQGGLATIHNCLWYIYGALLQQGGMYLPRADSGRLVVGTWWLVVLVVVTTYSGNLVAFLTFPKQEVPVTTVAELLDNRAFYTWSITKGSYMEMELKNSDEPKYIALLKGAELVTSSVGMGGAMASGSSLLQRVRLHRHAIIDWKLRLSYLMRGDRHEADNCDFSLSTEEFFHEKVAMIVPAGSPYLPVINKELDRMHKAGLIMRWLDAYLPKKDRCWKASSMMQEVNNHTVNLSDMQGSFFVLFMGFFSASTVLFLEFLYNRRKRRSEQIVIKPYVE

>AsegIR100d

MNIDNGLKLSALHKVQGARFISRRFFWDPGNVSNVMYIVMSENYQELERGLNKVTSDKFWNPTAVFIIVLQSYESYSLHDVTDLLHAFNIFYQVSLISRHSDDYAIYKYNFTKPDYCLKSGHLTFWSWCSDYYTEKNNLPLIYKGSIRNCRYKFITRNLWPFTNFDTSRRGSEQHFLSLFENQYGVKIELKEFGKVDKYGQLMNNLSMIMIQKVRNNEFEGAMAGYAITSENNRGNISYCYPLLIDHNFFVVAHSNFVEQWVAVLHQSPITCMVVGLLFVIFCVSAISLTIFPAGKDVSYNALTVLGYLLNKNYVRQIKSGGPQTLMFSSLLFTAFIIPYAIQANLFSVTTQPIRGFEPRSPQDLENYFPILFSEWHHRNEFPGHWDCGTRLRCLRIIKDSKSKNLYTVISEAHYRIYQWQIADDQCEMTTYRLREPFNSIYRTIFLRRGSVLIPYLDHFILQVSTTGIMQKFSRDIHSREWLKCKSHHRPEHVSLPLSNFYHVYMILVGGYCLSFVFFIYEVCSRSQRMRVLMRSNP

>AsegIR100i

MRAVYLLVFLLSTGTCFQIKIPGATFSQKLIECVRDIIAKYLKEAKEVTYIGNKVRDEELLKFINNANIVSVITKRGSTKQFVPHQAYLVSASNATYFAYRFFNLTRDPTWNPQARFLVIVRDLTEVDLKIMFDTFLKLHANNVLIVNATDDAHLYTYNPFDNYNCGKRYDTITSFGKCTQAHSLDLYPEKMVTGLQNCTFNVLMTQWPPYTILTTNESPKSPPALRNGVEPYLLRLIGELLGFDINLINDYGDADEFSTVSPDMQAVGLLKNIQDNVVDMYVSGMILLPSRAAAFSYIYGHLVYTDEIRFVVQRARNVRPWKNTYLEFELAVWLLLVLALVIYSILAIILLRAKDKSYVVLILLDNLVLHGRNITSRWAVKCFFMLWILFAYLVNTFYQSSLVSLTTNPAQEYQISTEEDINYYQLKPCLSSIMGRYYIESVQSNTEFDDSDACHRLMGSVHTVAKSDKLYTLILYGLYKYNEQDFFDEYGRPQVMPLPKPYSKVIYATYVYKGFPLIEKLCHKSIQLRESGLVGKVMSDMNYLKRITHSFHQRDFEGRLTIPWIIYVSGCTLSVITFAIEIILKRKQ

>AsegIR100j

MIVNGTTEAHLYTYNPFANYSCGKYYNDVYNLGLCSETTQNLYPNKLVTGLKNCTFKASVAHRPPFSINPAKIEGEKKLLGTEEYIFKVLSEKEQFKVISNFSYNADLYSSVEYNMTVSGPMMMLRNNETDIIFGGMMMVLTRAQALTWLCGYHDYNDELHFVVKRAEYVPIWKTVYIEFDPLVWFLLFVSFMVYFSMMVFLLRAKDKGYVALELLDNLLSHSRGIRTSMTLKYILIIWVWFAYLMNTFYQSSLVSLTTDPSKEYQVSTEEDLLEYEYKPCFAGSLRKYLATEHKHLTKKQVVPIDAPAIERCDTPIKALITVSRTPGMYSIVPRYTYLYYIRDIHDKWGNRLIYSFDKLYAKFLFGFYFYKGFPITHKLRLNALRIRENGLADKSIQDQYFLKTLKLRFSHKDFEVRFTLPWSVYVVGCAISIVAFLIEYLPKNRHRHHHQ
